# Nutritional Monitoring During Gender-Affirming Hormone Therapy: Body Composition and Metabolic Implications

**DOI:** 10.3390/nu18121967

**Published:** 2026-06-18

**Authors:** Martina Tosi, Fabrizia Lisso, Francesco Maruca, Carmelo Pujia, Taira Monge, Ersilia Troiano, Elisa Mazza

**Affiliations:** 1 Technical Scientific Association of Food, Nutrition and Dietetics (ASAND), 95128 Catania, Italy; martina.tosi@asand.it (M.T.); fabrizia.lisso@asand.it (F.L.); taira.monge@asand.it (T.M.); elisamazza@unicz.it (E.M.); 2 “G. Tatarella” Hospital, Foggia Local Health Authority, 71042 Cerignola, Italy; 3 Institute for Photonics and Nanotechnologies (CNR-IFN), 20133 Milano, Italy; francescomaruca9@gmail.com; 4 Clinical Nutrition, Renato Dubecco Hospital, 88100 Catanzaro, Italy; carmelopujia97@gmail.com; 5 San Luigi Gonzaga University Hospital, 10043 Orbassano, Italy; 6 Social Educational Directorate of Rome III Montesacro Municipality, 00139 Rome, Italy; 7 Department of Clinical and Experimental Medicine, University Magna Græcia, 88100 Catanzaro, Italy

**Keywords:** gender-affirming hormone therapy, transgender men, transgender women, body composition, metabolic adaptations, nutritional monitoring

## Abstract

**Background/Objectives**: Gender-affirming hormone therapy (GAHT) is associated with clinically relevant changes in body composition, energy metabolism, and functional capacity in transgender and gender-diverse individuals. The nutritional implications of these adaptations remain insufficiently characterized, and current assessment models, largely derived from cisgender populations, may not fully capture hormone-related body composition and metabolic changes. This narrative review aims to synthesize the metabolic and body composition effects of GAHT, evaluate methodological limitations in assessing nutritional status, and propose an integrated framework for clinical nutritional management. **Methods**: A narrative literature review was conducted through searches of PubMed/MEDLINE, Scopus, and Web of Science, complemented by screening of relevant guidelines and reference lists. Priority was given to longitudinal studies, mechanistic studies, systematic reviews, meta-analyses, and clinical guidance addressing GAHT-related changes in body composition, metabolism, nutritional status, and functional outcomes. **Results**: Available evidence suggests that GAHT is associated with sex steroid-related, tissue-specific changes in body composition and metabolism. In transgender men, testosterone is generally associated with increases in lean body mass (LBM), reductions in fat mass, and potential increases in visceral adiposity, alongside possible increases in energy expenditure and altered cardiometabolic profiles. In transgender women, estrogen therapy, combined with androgen suppression, is generally associated with reductions in LBM and redistribution of subcutaneous fat, with heterogeneous metabolic and functional responses. Across both groups, changes in body composition are not consistently reflected by the Body Mass Index or functional outcomes, suggesting a possible dissociation between structural and functional adaptation. Common assessment tools show limitations, including reliance on cisgender-derived reference standards and inability to capture dynamic hormonal transitions. **Conclusions**: Current evidence supports the need for a longitudinal and individualized interpretation of nutritional and body composition changes during GAHT. A shift toward longitudinal, multimodal nutritional assessment, integrating body composition, functional measures, biochemical markers, dietary intake, and clinical context, may improve clinical monitoring and reduce misclassification.

## 1. Introduction

Transgender and gender-diverse (TGD) individuals are people whose gender identity does not align with the sex assigned at birth [1,2]. Recent estimates indicate that approximately 25 million people worldwide identify as transgender, corresponding to roughly 0.3–0.6% of the global population. Access to gender-affirming services has steadily increased over recent decades, driven by greater social awareness, improved availability of specialized care, and the recognition of gender-affirming interventions as essential components of health care [3,4]. Gender-affirming care encompasses social, psychological, and medical interventions aimed at supporting gender congruence. Among these, gender-affirming hormone therapy (GAHT), based on the administration of estrogens, with or without anti-androgens or testosterone, represents the cornerstone of medical treatment and is now widely integrated into routine clinical practice worldwide [4]. Current standards and clinical guidelines provide structured recommendations for hormone initiation and monitoring while emphasizing the importance of individualized, multidisciplinary follow-up across the lifespan [4,5].

Although GAHT is primarily prescribed to induce desired secondary sex characteristics, sex steroids exert broad systemic effects that extend far beyond reproductive tissues. Testosterone and estrogens are key regulators of body composition and energy metabolism, modulating skeletal muscle protein turnover, adipose tissue distribution, resting energy expenditure, insulin sensitivity, and bone remodeling [6,7,8]. The sexual dimorphism observed in cisgender populations, characterized by greater lean body mass (LBM) and higher metabolic rates in males and higher relative fat mass with distinct regional deposition in females, reflects the central role of these hormones in shaping metabolic phenotype [9]. Consistent with these mechanisms, GAHT induces substantial remodeling of fat mass, fat-free mass, total body water, and bone mineral density [10,11,12].

Longitudinal studies consistently report increases in LBM and reductions in fat mass in transgender men treated with testosterone, whereas transgender women receiving estrogens, with or without anti-androgens, typically exhibit the opposite pattern, frequently accompanied by a redistribution of adiposity toward a more gynoid profile [13,14]. These changes are not merely anthropometric but may translate into meaningful alterations in energy requirements, physical function, and long-term cardiometabolic health. Despite these clinically relevant adaptations, the nutritional and body composition aspects of transgender health remain underexplored. Most research has focused on endocrinological safety and psychosocial outcomes, while fewer studies have examined nutritional status, metabolic needs, or the validity of conventional body composition tools during hormonal transition. As a result, current evidence remains fragmented and insufficient to guide standardized clinical monitoring.

At the same time, several considerations indicate that careful nutritional and metabolic evaluation is particularly warranted in this population. Hormone-induced shifts in lean and fat compartments may modify energy balance and macronutrient requirements, while alterations in fat distribution and insulin sensitivity could influence cardiometabolic and hepatic risk trajectories [15,16,17]. Bone health, muscle function, and micronutrient status represent additional areas of concern [6,7,12]. Moreover, higher rates of body image dissatisfaction and disordered eating behaviors reported among transgender individuals may further complicate the assessment and management of nutritional status [18,19,20]. Together, these factors highlight the need for an integrated approach that links metabolic physiology, body composition assessment, and nutritional care within gender-affirming treatment pathways. In this context, nutritional assessment and dietary management should be considered integral components of gender-affirming care rather than ancillary aspects.

Given these challenges, a comprehensive and critical synthesis of current knowledge is needed to bridge the gap between endocrinological management and nutritional science. Importantly, current nutritional models, largely derived from cisgender populations, may not fully capture the changes in body composition, metabolism, and functional status that occur during GAHT, creating uncertainty in dietary assessment and clinical monitoring. Addressing this gap requires longitudinal and individualized approaches capable of integrating hormonal exposure, metabolic markers, body composition, dietary intake, and functional outcomes over time.

Therefore, the aim of this narrative review is threefold: (i) to summarize the metabolic and body composition effects of GAHT; (ii) to analyze methodological challenges in assessing nutritional status and body composition in TGD individuals; and (iii) to provide practical clinical recommendations and an integrated framework for evidence-based monitoring and personalized nutritional management.

## 2. Materials and Methods

This narrative review was conducted to synthesize current evidence on nutritional status, body composition, metabolic adaptations, and assessment strategies in transgender and gender-diverse individuals undergoing GAHT. The literature searches were performed in PubMed/MEDLINE, Scopus, and Web of Science from database inception to June 2026. Search terms included combinations of: “gender-affirming hormone therapy”, “transgender men”, “transgender women”, “transgender and gender-diverse”, “body composition”, “lean body mass”, “fat mass”, “visceral adipose tissue”, “bioelectrical impedance analysis”, “DXA”, “resting energy expenditure”, “nutritional status”, “micronutrients”, “muscle strength”, and “functional assessment”.

Reference lists of relevant articles, systematic reviews, meta-analyses, and clinical guidelines were also screened. Priority was given to longitudinal studies, mechanistic studies, clinical studies, systematic reviews, meta-analyses, and guidelines addressing GAHT-related changes in body composition, metabolism, nutritional status, skeletal health, and functional outcomes. Studies not focused on GAHT, articles without relevance to nutritional or body composition outcomes, non-clinical commentaries without original or interpretative value, and studies without an available full text were excluded. Given the narrative nature of this review, no formal risk-of-bias assessments or quantitative meta-analyses were performed.

## 3. Metabolic Effects of Gender-Affirming Hormone Therapy: Sex Steroid Physiology—Divergent Trajectories in Transgender Men and Transgender Women

GAHT induces coordinated metabolic adaptations that extend beyond phenotypic changes, affecting skeletal muscle, adipose tissue, glucose–insulin homeostasis, and lipid metabolism. Because GAHT in transgender men (TM) involves testosterone exposure, whereas, in transgender women (TW), it combines estrogen therapy with androgen suppression, metabolic trajectories diverge in direction and magnitude, particularly during the first 6–18 months, when body composition changes are most pronounced [13,14].

To facilitate interpretation of the available evidence, Table 1 summarizes the main GAHT-related body composition, metabolic, functional, and skeletal effects reported in transgender men and transgender women. Given the heterogeneity of available studies, the table reports the direction of change and available quantitative estimates where applicable.

### 3.1. Transgender Men: Testosterone-Driven Anabolic Remodeling, Tissue-Specific Metabolic Reprogramming, and Emerging Metabolic Complexities

GAHT in TM, based on exogenous testosterone administration, induces a coordinated and time-dependent remodeling of body composition and metabolic function that extends beyond phenotypic masculinization. Rather than representing a static shift toward a male physiological profile, these adaptations reflect a dynamic process of tissue-specific metabolic reprogramming involving skeletal muscle, adipose tissue, and energy metabolism. A central component of this remodeling is the expansion of lean body mass (LBM), primarily driven by enhanced skeletal muscle anabolism. Testosterone activates androgen receptor-mediated signaling pathways, including PI3K–Akt–mTOR, promoting protein synthesis and inhibiting proteolysis, resulting in net protein accretion [7]. Quantitative evidence from a recent meta-analysis indicates a mean increase in LBM of approximately +4.98 kg, accompanied by a reduction in fat mass of −2.13 kg within the first year of therapy, although with low-to-moderate certainty due to study heterogeneity [22]. These changes occur predominantly within the first 6–12 months and tend to plateau thereafter, suggesting a phase-dependent trajectory [13,22]. Importantly, recent longitudinal data indicate that increases in LBM are accompanied by improvements in muscle strength and physical performance, although the magnitude of functional adaptation may vary according to age and baseline status [12,23]. However, emerging evidence suggests that changes in muscle strength and performance are not always proportional to increases in LBM, highlighting the importance of muscle quality and neuromuscular adaptation as additional determinants of functional outcomes. In addition, LBM remains a quantitative measure and does not directly reflect muscle quality or functional capacity. Strength adaptation may also depend on neuromuscular activation, motor unit recruitment, muscle architecture, fiber composition, intramuscular lipid infiltration, and baseline training status [12,23,31,32]. Therefore, testosterone-related gains in LBM should be interpreted together with functional outcomes, physical activity, and nutritional adequacy, rather than as an isolated marker of muscle adaptation.

Beyond muscle anabolism, testosterone induces a complex reorganization of adipose tissue compartments. While total fat mass generally decreases, emerging evidence indicates that this reduction may coexist with a relative increase in visceral adipose tissue (VAT), highlighting a divergence between global adiposity and regional fat distribution. In a recent longitudinal study, Larose et al. reported a significant increase in VAT after 12 months of testosterone therapy, associated with an unfavorable metabolic profile despite stable inflammatory markers [23]. Similarly, longitudinal MRI-based studies confirm that GAHT is associated with compartment-specific changes in adipose tissue and ectopic fat deposition, particularly at the hepatic level, reinforcing the concept that hormonal therapy reshapes rather than uniformly reduces adiposity [13,16,24]. These compositional changes are closely linked to modifications in energy metabolism. The expansion of metabolically active lean tissue is expected to increase resting energy expenditure (REE), contributing to shifts in energy balance and nutrient requirements. Although direct measurements of REE in TM remain limited, available longitudinal data support a progressive increase during early treatment phases [8,16]. In parallel, testosterone appears to influence substrate utilization, with some studies suggesting enhanced lipid oxidation and altered glucose metabolism [15,26]. However, findings on insulin sensitivity remain heterogeneous. For instance, a recent prospective study in transgender adolescents reported significant changes in body composition and bone parameters without corresponding alterations in insulin sensitivity over 12 months of testosterone therapy, suggesting that metabolic responses may vary according to age, baseline metabolic status, and duration of exposure [26].

From a cardiometabolic perspective, testosterone therapy induces a shift toward a lipid profile more typically observed in cisgender males (CMs), characterized by reductions in HDL cholesterol and variable changes in LDL cholesterol and triglycerides [27,49]. When considered alongside the preferential redistribution of fat toward a visceral compartment, these changes suggest a complex metabolic trade-off, in which favorable anabolic effects coexist with potential cardiometabolic risks. Additional studies report increases in hematocrit and blood pressure during masculinizing GAHT, further supporting the need for careful longitudinal monitoring [27,29,49]. Testosterone also exerts significant effects on skeletal metabolism. While GAHT in TM is generally associated with the maintenance or increase in bone mineral density (BMD), recent prospective data suggest a more nuanced picture. Early increases in bone turnover markers, such as C-terminal telopeptide (CTX) and P1NP, have been observed within months of therapy initiation, indicating active skeletal remodeling [34]. Moreover, longitudinal studies have reported site-specific responses, with stable BMD at the lumbar spine and total hip but modest reductions at the femoral neck after one year of therapy, particularly influenced by age [35]. These findings suggest that skeletal adaptations to testosterone are not uniform and may depend on both biological and clinical factors, including nutritional status and treatment adherence.

From a nutritional and clinical standpoint, these hormonally driven adaptations have several important implications. The increase in LBM and the associated rise in energy expenditure suggest a recalibration of energy requirements and potentially higher protein needs to support muscle accretion and maintenance, as supported by the relationship between fat-free mass and resting energy expenditure, as well as evidence on androgen-mediated muscle anabolism [6,7,42]. At the same time, the redistribution of adipose tissue toward a more visceral phenotype and the observed changes in lipid profile emphasize the importance of dietary quality, particularly fat composition, in modulating cardiometabolic risk [27,43].

From a clinical perspective, these findings indicate that nutritional strategies should be aligned with the temporal dynamics of hormonal adaptation rather than based on static assumptions of metabolic demand [4,5]. Importantly, these changes should not be interpreted solely as isolated alterations in body composition but rather as components of a dynamic energy balance system. The increase in LBM and resting energy expenditure, if not accompanied by a proportional adaptation in energy intake, may lead to negative energy balance, potentially limiting optimal muscle accretion, in line with the dependence of muscle protein synthesis on energy and protein availability [6,7,42]. Conversely, excess caloric intake during phases of reduced anabolic sensitivity may favor disproportionate fat accumulation, particularly in the visceral compartment, as suggested by evidence linking positive energy balance and ectopic fat deposition to metabolic risk [16,27]. This highlights the risk of a mismatch between hormonal adaptation and dietary intake, underscoring the importance of individualized and phase-specific nutritional strategies during GAHT [27].

Overall, testosterone therapy in transgender men induces a complex and dynamically evolving metabolic phenotype, characterized by anabolic effects on skeletal muscle, redistribution of adipose tissue, and modulation of energy and substrate metabolism. While many of these adaptations align with desired physical outcomes, emerging evidence highlights the presence of metabolic complexities and potential trade-offs that challenge simplified interpretations. These findings support the need for integrated, physiology-informed, and nutrition-oriented approaches to the assessment and management of individuals undergoing gender-affirming treatment [4,5,27].

### 3.2. Transgender Women: Estrogen-Driven Adipose Remodeling, Muscle–Fat Cross-Talk, and Heterogeneous Metabolic Trajectories

GAHT in TW, typically based on estrogen administration combined with androgen suppression, induces a complex and multi-system metabolic remodeling that cannot be adequately described as a simple inverse mirror of testosterone-driven adaptations. Rather than following a uniform or linear trajectory, the feminizing hormonal milieu generates a heterogeneous phenotype characterized by coordinated yet partially discordant changes across skeletal muscle, adipose tissue, liver, and cardiometabolic pathways. These adaptations are influenced by treatment duration, route of estrogen administration, degree of androgen suppression, and baseline metabolic status. A defining feature of this process is the progressive reduction in LBM, primarily driven by androgen deprivation and attenuation of anabolic signaling. Reduced androgen receptor activation leads to diminished stimulation of muscle protein synthesis, potentially shifting the balance toward net protein loss. Longitudinal studies consistently report reductions in LBM within the first year of therapy [11,13,22], and recent prospective evidence indicates that these compositional changes are accompanied by measurable declines in muscle strength and physical performance [12,21,22]. However, emerging data indicate that reductions in muscle mass are not always directly proportional to declines in functional performance, suggesting a dissociation between muscle quantity and muscle quality, with neuromuscular and metabolic factors contributing to functional outcomes [30,31,32]. This functional dimension suggests that feminizing GAHT may be associated with an early reduction in metabolic and physical reserve, particularly in the absence of targeted lifestyle interventions.

In parallel, estrogen exposure promotes adipose tissue expansion through enhanced adipocyte differentiation, lipid storage, and regional redistribution. While preferential accumulation of subcutaneous adipose tissue (SAT) remains a hallmark of feminizing therapy, changes in VAT are highly variable and appear to be time dependent. Short-term studies suggest a reduction in the VAT/SAT ratio, whereas longer-term observations report stable or even increased VAT in some individuals [11,13,14,24,25]. Importantly, ectopic fat depots may respond divergently to hormonal modulation, with reductions in intrahepatic lipid content reported despite increases in circulating triglycerides, supporting a compartment-specific metabolic response [16,24]. These findings reinforce selective adipose redistribution rather than uniform fat gain. These structural changes occur within a broader network of metabolic adaptations involving muscle–fat cross-talk and substrate metabolism. The reduction in metabolically active lean tissue likely contributes to lower REE during feminizing GAHT. However, changes in energy expenditure may not be explained by LBM loss alone, as estrogen exposure and androgen suppression may also influence mitochondrial function, substrate utilization, lipid oxidation, and insulin-mediated glucose handling [8,15,16,17,26].

However, findings on insulin sensitivity remain inconsistent, with studies reporting reduced insulin sensitivity or minimal changes [15,26,27]. This heterogeneity suggests that metabolic responses are not solely determined by body composition but by complex hormonal and tissue interactions.

From a cardiometabolic perspective, feminizing GAHT is associated with a multifaceted risk profile, including increases in triglycerides, alterations in cholesterol fractions, and pro-thrombotic changes [27,49]. These metabolic alterations do not consistently correlate with body composition, limiting the utility of anthropometric measures alone for risk assessment.

Bone metabolism further exemplifies the complexity of estrogen effects in this population. While estrogen is known to exert protective effects on bone, transgender women may present with suboptimal bone mineral density (BMD) at baseline and show modest site-specific improvements during GAHT, influenced by age and adherence [35,36,37].

From a nutritional perspective, the metabolic phenotype in TW introduces bidirectional challenges. Reduced LBM and energy expenditure may predispose to positive energy balance if intake is not adjusted, increasing fat accumulation. Conversely, insufficient energy and protein intake may exacerbate LBM loss. Preservation of muscle mass therefore becomes a key objective, highlighting the importance of protein intake and resistance-based physical activity. At the same time, increased fat mass and altered lipid profiles emphasize dietary quality, particularly fat composition [27,43].

From a clinical perspective, these adaptations should be interpreted within an individualized energy balance framework aligned with the treatment phase. The coexistence of reduced LBM, increased adiposity, and heterogeneous metabolic responses creates a scenario in which both positive and negative energy imbalances may have clinically relevant consequences, reinforcing the importance of phase-specific nutritional strategies [27].

Feminizing hormone therapy in transgender women results in a complex and heterogeneous metabolic landscape, characterized by adipose tissue expansion, reduction in LBM, and variable functional outcomes. These findings support the need for integrated, physiology-informed, and nutrition-oriented approaches to gender-affirming care [4,5,27]. Consideration of aging-related endocrine changes and long-term hormone exposure is also relevant in this context, particularly with respect to bone health, as highlighted in the recent literature on transgender health and aging [38].

## 4. Assessment Challenges in Body Composition and Nutritional Status

The assessment of nutritional status and body composition in transgender and gender-diverse individuals undergoing GAHT presents substantial methodological and clinical challenges. These challenges arise directly from the complex, dynamic, and tissue-specific adaptations described above, which are not adequately captured by conventional assessment models. As a result, traditional anthropometric and body composition methods may fail to detect clinically relevant changes in tissue compartments, muscle quality, and metabolic risk, increasing the likelihood of misclassification and suboptimal clinical interpretation. Importantly, these adaptations may occur independently of changes in total body weight or body mass index (BMI). Recent evidence indicates that, even in the presence of stable body weight, significant alterations may occur in fat-free mass, body cell mass, phase angle, visceral adiposity, and functional performance [50,51,52,53]. These findings underscore the limitations of relying on single or static indicators and support the need for a multidimensional and longitudinal assessment approach.

### 4.1. Limitations of BMI

BMI remains one of the most widely used indicators of nutritional status in both clinical and research settings. However, its applicability in transgender populations undergoing GAHT is particularly limited. BMI does not distinguish between fat mass and LBM, nor does it capture regional fat distribution, muscle quality, or functional status, making it poorly suited to hormonally driven body composition remodeling. During GAHT, individuals may experience marked and divergent shifts in fat-free mass and fat mass without corresponding variations in BMI. Transgender men typically exhibit increases in LBM alongside reductions in fat mass, whereas transgender women show the opposite pattern, often accompanied by reductions in muscle strength and functional capacity [11,13,14,22,24]. As a result, BMI may remain stable despite clinically relevant alterations in body composition and metabolic profile.

This limitation is further supported by longitudinal evidence. Studies in transgender women undergoing feminizing GAHT demonstrate that BMI may remain unchanged while significant reductions occur in fat-free mass, fat-free mass index, body cell mass, and phase angle, parameters closely associated with nutritional status and cellular integrity [50]. These findings indicate that BMI may substantially underestimate hormonally induced changes in both structural and functional compartments.

Moreover, systematic reviews and meta-analyses confirm that GAHT is associated with significant changes in body composition parameters, while highlighting heterogeneous findings and low-to-moderate certainty of evidence [15,22]. This reinforces the need for cautious interpretation of BMI-based findings and limits its use as a primary outcome measure in this population.

A further limitation of BMI lies in its inability to capture compartment-specific adipose tissue remodeling. GAHT induces complex and sometimes discordant changes in visceral and subcutaneous fat depots, which have distinct metabolic implications. Individuals may therefore remain within the same BMI category while experiencing increases in visceral adiposity or deterioration in body composition quality, changes that are more strongly associated with cardiometabolic risk than total body weight alone [14,16].

An additional and critical issue concerns the use of BMI cut-offs derived from cisgender populations. These thresholds are based on reference models that do not account for hormone-induced shifts in body composition or the transitional metabolic phenotypes observed during GAHT. More broadly, this reflects a systemic limitation affecting multiple clinical assessment tools. Emerging evidence highlights that commonly used clinical algorithms may fail to incorporate key variables, such as hormonal regimen, duration of therapy, and sex assigned at birth, thereby limiting their applicability in transgender individuals [54,55].

Taken together, these considerations indicate that BMI, while simple, widely accessible, and still useful as a broad epidemiological marker of cardiometabolic risk, provides an incomplete representation of nutritional status and body composition during GAHT. Its main limitation is its inability to characterize fat and lean compartments, regional adiposity, muscle quality, and functional status. Therefore, BMI should not be used as a standalone phenotyping tool but should be interpreted within an integrated framework that includes direct measures of body composition, metabolic parameters, and functional outcomes.

### 4.2. Anthropometric Assessment: Regional Phenotyping, Peripheral Markers, and the Role of Skinfolds

Anthropometric assessment represents a practical, low-cost, and widely accessible approach for evaluating regional body characteristics and remains an important component of clinical monitoring in transgender individuals undergoing GAHT. Its primary value lies in the characterization of regional phenotypes, particularly the distribution of adipose and peripheral tissues, which are profoundly influenced by sex steroid exposure [54,56]. Core anthropometric measures include waist circumference, hip circumference, and waist-to-hip ratio (WHR), which are commonly used as proxies of fat distribution and cardiometabolic risk [14,57]. These parameters are particularly relevant during GAHT, where hormonal modulation induces complex and often incomplete redistribution of adipose tissue. As a result, individuals frequently exhibit intermediate or non-binary fat distribution patterns, which do not fully align with cisgender reference phenotypes, thereby limiting the interpretability of conventional thresholds [22,25].

Beyond central adiposity, peripheral anthropometric measures, such as mid-upper-arm circumference and calf circumference, provide information on peripheral tissue compartments and are often used as surrogate indicators of muscle mass and nutritional status [54,58,59]. However, evidence suggests that changes in limb circumferences during GAHT may be modest and may not fully reflect underlying alterations in muscle mass or composition. This highlights a potential dissociation between regional anthropometry and whole-body compositional adaptation, particularly in hormonally driven muscle remodeling [24,25]. In response to these limitations, increasing attention has been directed toward integrated anthropometric indices that combine central and peripheral measurements. For instance, the waist-to-calf circumference ratio (WCR) has emerged as a marker capturing the balance between central adiposity and peripheral muscle mass, and has been associated with cardiometabolic risk in the general population [59,60]. Although not yet validated in transgender populations, such indices may offer added value in GAHT, where concurrent changes in fat distribution and lean tissue complicate single-site interpretation.

Skinfold thickness measurement represents an additional component of anthropometric assessment, providing a more direct estimate of subcutaneous adipose tissue at specific anatomical sites (e.g., triceps, subscapular, suprailiac, abdominal, and thigh). Unlike circumferences, which reflect combined contributions of fat, muscle, and bone, skinfolds allow a more compartment-specific evaluation of subcutaneous fat distribution [61,62,63]. Despite these advantages, skinfold assessment presents several methodological limitations. Its accuracy is influenced by operator expertise, caliper type, measurement site, and tissue-specific factors, such as skin elasticity, obesity, and regional fat redistribution [61,63]. Reproducibility may be reduced in individuals with substantial adiposity or marked changes in fat distribution. Moreover, even in cisgender populations, skinfold-derived predictive equations show considerable inter-equation variability and reduced accuracy at extremes of body composition. This further limits their validity in transgender individuals undergoing GAHT, whose body composition may change over time and may not conform to cisgender-derived assumptions [62].

Furthermore, device-related factors, such as caliper type and applied pressure, may introduce measurement variability, potentially affecting longitudinal comparability [61]. This is particularly relevant in GAHT, where relatively small but clinically meaningful changes in subcutaneous fat thickness may occur and require high measurement precision.

Taken together, anthropometric assessment should be considered a valuable but indirect tool for evaluating body composition. Its strengths lie in feasibility, accessibility, and the ability to track regional changes over time. However, interpretation in transgender individuals undergoing GAHT requires careful contextualization due to hormone-induced tissue redistribution, lack of population-specific reference standards, and methodological constraints [15,24]. Accordingly, anthropometry should be integrated within a multimodal and longitudinal assessment framework rather than used as a standalone diagnostic approach.

### 4.3. Bioelectrical Impedance Analysis: Accessibility, Algorithm Dependency, and Interpretative Fragility

Bioelectrical impedance analysis (BIA) is widely used in clinical practice due to its portability, low cost, and ease of use, allowing rapid estimation of body composition parameters, including fat mass, fat-free mass, total body water, and derived indices, such as phase angle and body cell mass. However, it should be emphasized that BIA provides model-derived estimates rather than direct measurements of body composition. Although validated in general populations and showing acceptable agreement with reference methods such as dual-energy X-ray absorptiometry (DXA) under standardized conditions, its accuracy is significantly influenced by physiological assumptions that may not hold in populations characterized by altered body composition or fluid distribution [53,64].

In transgender individuals undergoing GAHT, BIA presents substantial methodological and interpretative challenges. A central limitation is its reliance on predictive equations derived from cisgender populations, embedding binary sex-specific assumptions within device algorithms. In individuals undergoing hormonal transition, this leads to a problem of algorithm dependency. Evidence shows that BIA-derived estimates may vary substantially depending on whether the device is set to “male” or “female,” producing systematic differences in fat mass and fat-free mass estimations [51,52,53]. Similarly, comparisons with DXA demonstrate reduced agreement when sex-specific equations are not aligned with the individual’s physiological profile [53].

Beyond algorithm dependency, BIA is intrinsically sensitive to hydration status and fluid distribution, which directly influence electrical conductivity independently of tissue composition. Sex steroids modulate extracellular and intracellular water compartments, thereby affecting impedance measurements through non-compositional mechanisms. Hydration variability is therefore a major source of systematic error in impedance-derived estimates [64]. In the context of GAHT, this introduces a critical interpretative challenge, as observed changes in fat-free mass may partially reflect fluid redistribution rather than true alterations in lean tissue.

Recent longitudinal studies further highlight the complexity of interpreting BIA-derived parameters in transgender populations. In TW undergoing feminizing GAHT, reductions in fat-free mass, body cell mass, and phase angle have been reported over the first year of treatment, even in the absence of significant changes in BMI [50]. While phase angle is increasingly considered a marker of cellular integrity and nutritional status, its interpretation remains uncertain due to the absence of population-specific reference standards [42,50].

Importantly, emerging evidence suggests that BIA-derived changes cannot be interpreted in isolation from functional and endocrine context. Studies integrating body composition and functional outcomes indicate that changes in LBM are not consistently proportional to changes in physical performance, reflecting interactions between hormonal exposure, neuromuscular adaptation, and tissue remodeling [24,25]. Moreover, recent findings suggest associations between BIA-derived parameters and circulating hormone levels, indicating that these measures may reflect ongoing endocrine modulation rather than stable physiological states [53].

Taken together, these considerations highlight a key limitation of BIA in this setting, as its outputs are not purely empirical measurements but rather model-derived estimates influenced by algorithmic assumptions, hydration status, and hormonal environment. This intrinsic interpretative fragility makes BIA particularly vulnerable to misclassification when used as a standalone tool for assessing nutritional status or metabolic risk in transgender individuals undergoing GAHT [10,65].

At present, no transgender-specific BIA predictive equations or validated algorithmic settings are available for routine clinical use. Evidence indicates that estimates differ substantially according to binary device settings, supporting the use of BIA primarily for standardized longitudinal within-subject monitoring rather than cross-sectional classification [52,53].

In clinical practice, BIA should therefore be regarded as a feasible but inherently limited method. Its strengths lie in accessibility and suitability for longitudinal monitoring, whereas its limitations concern validity and interpretability [66]. Accordingly, BIA should be integrated within a multimodal and longitudinal assessment framework, alongside more robust compositional methods, such as DXA, functional measures, and clinical context, to accurately capture the complex adaptations occurring during gender-affirming treatment.

### 4.4. Dual-Energy X-Ray Absorptiometry: Measurement Strength Versus Interpretative Bias

DXA is widely regarded as the reference method for body composition assessment in both clinical and research settings, providing detailed estimates of total and regional fat mass, lean soft tissue, and bone mineral density with high reproducibility. In transgender individuals undergoing GAHT, DXA is particularly valuable for detecting compartment-specific and longitudinal changes that cannot be captured by anthropometric indices alone. Recent studies support its central role in monitoring changes in LBM, adiposity, and regional body composition during GAHT [21,51].

A key strength of DXA lies in its ability to characterize regional and compartment-specific body composition. Unlike global indices, DXA differentiates appendicular LBM, trunk fat mass, and android versus gynoid fat distribution, enabling detailed assessment of tissue remodeling across body regions. This is particularly relevant in GAHT, where hormonal exposure induces non-uniform and region-specific adaptations.

In TM, testosterone therapy promotes increases in appendicular LBM, reflecting skeletal muscle accretion predominantly in the limbs, while total and regional fat mass decrease. In TW, estrogen-based therapy is associated with reductions in LBM, particularly in upper-body compartments, and increases in total and regional fat mass, with preferential accumulation in gynoid regions, such as hips and thighs [11,33]. Importantly, these changes are not homogeneous across body regions, with differential adaptations between trunk and appendicular compartments, highlighting the limitations of whole-body estimates [11]. DXA-derived estimates of visceral adipose tissue further illustrate this complexity. Although average changes in visceral fat are often modest, substantial interindividual variability is consistently observed, with trajectories that are not necessarily proportional to total fat mass changes [11,21].

However, the main limitation of DXA in this context lies less in measurement accuracy than in clinical interpretation. DXA outputs are interpreted using reference frameworks derived from cisgender populations and based on sex-specific assumptions that may not adequately reflect transitional phenotypes during GAHT [54,55]. This is particularly relevant for parameters such as appendicular LBM, sarcopenia indices, and bone mineral density, where identical values may lead to different clinical classifications depending on the reference standard applied. Accordingly, individuals undergoing GAHT may occupy intermediate or discordant positions relative to cisgender reference groups, complicating risk stratification and diagnosis [58].

Similar interpretative challenges apply to skeletal assessment. Although DXA-derived bone mineral density is central to bone health evaluation, case-based evidence has reported discrepancies in interpretation in transgender and gender-diverse individuals, particularly in younger patients, as well as at specific skeletal sites, such as the forearm. These discrepancies reflect not only biological variability in GAHT-related bone remodeling but also the limitations of applying cisgender-derived reference criteria to this population [36,55].

Beyond structural assessment, DXA does not capture functional or metabolic tissue properties. Longitudinal evidence shows that changes in LBM are not consistently proportional to changes in muscle strength or physical performance, indicating a dissociation between structural and functional adaptation [12,30]. Similarly, tissues with comparable DXA-derived changes may differ substantially in metabolic activity and clinical relevance depending on their regional or ectopic distribution. Therefore, DXA provides quantitative but not qualitative information on tissue function. Taken together, DXA represents a highly robust and informative measurement tool, but not a self-sufficient interpretative framework. Its clinical value depends on integration with hormonal status, treatment duration, functional assessment, and metabolic markers. Accordingly, DXA should be viewed as one component of a broader, multimodal assessment strategy rather than a standalone diagnostic solution for individuals undergoing GAHT [39,40,41].

### 4.5. Muscle Strength and Functional Assessment: Dissociation, Heterogeneity, and Clinical Integration

Assessment of muscle strength and physical function represents a critical component of nutritional and metabolic evaluation in transgender individuals undergoing GAHT. While body composition techniques quantify changes in LBM, they do not fully capture the functional outcomes. Evidence increasingly shows that structural and functional adaptations to GAHT may follow partially independent trajectories, highlighting a clinically relevant dissociation between muscle mass and performance. Among available tools, handgrip strength is the most widely used and validated measure of muscle function. It is simple, reproducible, and strongly associated with overall muscle strength, physical performance, and long-term health outcomes. In transgender populations, it provides a direct assessment of functional adaptation that is independent of body composition estimates. Longitudinal evidence shows that testosterone therapy increases handgrip strength, with a clear age-dependent response, including greater gains in younger individuals and attenuated effects in those initiating treatment later in life [12]. Similarly, masculinizing GAHT increases maximal muscle strength by approximately 7–12%, while muscle power appears largely unchanged; conversely, feminizing GAHT leads to reductions in both strength and power, particularly within the first year of treatment [31]. These findings indicate that different components of muscle function (force and power) may respond differently to hormonal modulation.

Importantly, changes in muscle strength are not strictly proportional to changes in LBM. A systematic review including over 1200 transgender individuals confirmed that masculinizing GAHT generally increases strength and feminizing GAHT decreases it, but with substantial interindividual variability influenced by treatment modality, duration, and physical activity [31,32]. This variability suggests that muscle function reflects not only muscle quantity but also neuromuscular efficiency, fiber composition, and metabolic factors. Consistently, longitudinal data show that in TW, upper-body strength declines alongside LBM, whereas lower-body strength may be relatively preserved, indicating region-specific adaptation and incomplete functional convergence toward cisgender female profiles [30].

Beyond handgrip strength, lower-extremity tests for chair–stand and gait speed performance may provide clinically relevant information on mobility and functional reserve [67]. This is particularly important because GAHT-related changes may differ between upper- and lower-body compartments [21]. Although performance may partially approach that of cisgender individuals of the affirmed gender over time, evidence remains limited, especially in long-term and non-athletic populations [30].

Interpretation of muscle strength in transgender individuals is further complicated by the lack of appropriate reference standards. Normative values for handgrip strength and performance tests are derived from cisgender populations and stratified by sex, making their application during GAHT problematic. Individuals often present intermediate functional phenotypes that cannot be adequately classified using binary thresholds. For this reason, longitudinal within-subject monitoring is particularly valuable. Tracking individual trajectories over time allows assessment of functional adaptation independently of sex-based cut-offs and enables identification of clinically relevant deviations, such as inadequate strength gains, excessive decline, or dissociation between muscle mass and function. Taken together, muscle strength assessment provides essential and non-redundant clinical information and should be considered a core component of evaluation. Muscle function reflects the integrated effects of endocrine, metabolic, and neuromuscular adaptation and adds a dimension that cannot be inferred from body composition alone. Its systematic inclusion is therefore essential for a comprehensive and physiologically meaningful assessment of health and functional status during GAHT.

### 4.6. Reference Standards: Sex Versus Gender and the Challenge of Classification

A major unresolved challenge in the assessment of nutritional status, body composition, and skeletal health in transgender individuals undergoing GAHT is the lack of appropriate reference standards. Most currently used thresholds for body composition, sarcopenia, bone mineral density (BMD), and fracture risk are derived from cisgender populations and remain anchored to binary sex classification systems. However, GAHT induces dynamic and often partial shifts in body composition and tissue distribution, generating phenotypes that may not align with either cisgender male or female reference models. As a result, classification becomes highly dependent on the chosen reference framework rather than reflecting underlying physiology [12,58,68]. In practical terms, this means that the same DXA- or strength-derived value may lead to different clinical classifications depending on the reference system applied, with direct implications for the diagnosis of sarcopenia, metabolic risk, or bone fragility.

This limitation is particularly evident in muscle-related outcomes. Recent data in TW suggest an intermediate phenotype, with lower muscle mass than cisgender men but higher values than cisgender women, accompanied by reduced strength and a potential risk profile compatible with early sarcopenic vulnerability [31,32]. Consequently, identical appendicular LBM values may be classified as normal or low depending on whether male or female reference cut-offs are applied, leading to discordant sarcopenia classification and risk stratification [58]. It should be noted that contemporary sarcopenia definitions already integrate muscle strength, muscle quantity/quality, and physical performance. However, their application during GAHT remains challenging because these domains may change asynchronously and are still interpreted using cisgender-derived reference thresholds.

A similar interpretative issue applies to skeletal assessment. Case-based evidence [55] highlighted clinically relevant discrepancies in DXA interpretation in transgender and gender-diverse individuals, particularly in younger patients, as well as at specific skeletal sites, such as the forearm, highlighting that the problem lies not only in measurement but in the application of conventional interpretative frameworks to populations for whom they were not designed [58]. Recent observational data further indicate that baseline differences in bone density and body composition are already present before GAHT initiation, suggesting that misclassification may occur even prior to hormonal treatment when cisgender reference standards are used [69].

Recent evidence also questions the validity of fracture risk prediction tools in this context. A 2025 prospective study [36] reported that skeletal responses to GAHT vary according to age and sex assigned at birth, highlighting that FRAX algorithms are based on cisgender reference data and do not account for hormonal regimen, duration of therapy, or sex assigned at birth. Accordingly, FRAX-based estimates may systematically over- or underestimate fracture risk depending on the selected sex category, supporting the need for dedicated tools in this population [36].

At the guideline level, current official densitometry recommendations also reveal how provisional and imperfect existing solutions remain. The 2023 ISCD Official Positions [68] recommend using a uniform female normative database for T-score calculation in transgender and gender-non-conforming individuals aged ≥50 years, while suggesting Z-scores be derived from the database aligned with gender identity. Although pragmatic, this approach may still misestimate risk in individuals with incomplete or early physiological transition and underscores the absence of validated transgender-specific reference systems. It also highlights the tension between biological comparators, gender-affirming practice, and longitudinal hormonal adaptation.

These challenges may be even greater in younger populations. Recent works in transgender youth question the assumption that BMD Z-scores trajectories may not follow expected patterns in the absence of intervention, indicating that developmental reference models may be insufficient [37,70]. In addition, high interindividual variability in body composition and fat distribution trajectories during GAHT further complicates the use of fixed age- and sex-based thresholds in adolescents and young adults [21]. Taken together, these issues indicate that the problem of reference standards in transgender health is both technical and conceptual. The choice between sex-assigned and gender-affirmed reference systems forces clinicians to apply categorical frameworks that only partially reflect hormonally mediated biological reality. Instead of selecting between imperfect standards, a more appropriate strategy is to prioritize longitudinal within-subject monitoring and phenotype-informed interpretation, moving toward GAHT-specific or transition-aware reference frameworks.

Until such standards are developed, clinical practice should rely on three principles: (i) consistent use of the same reference system over time; (ii) integration of compositional, functional, and metabolic data; and (iii) interpretation of results within the individual clinical trajectory rather than through isolated thresholds. Overall, body composition and skeletal indices should always be interpreted cautiously, taking into account hormonal status, treatment duration, developmental stage, and clinical context [36,55,58,68].

**Figure 1:**
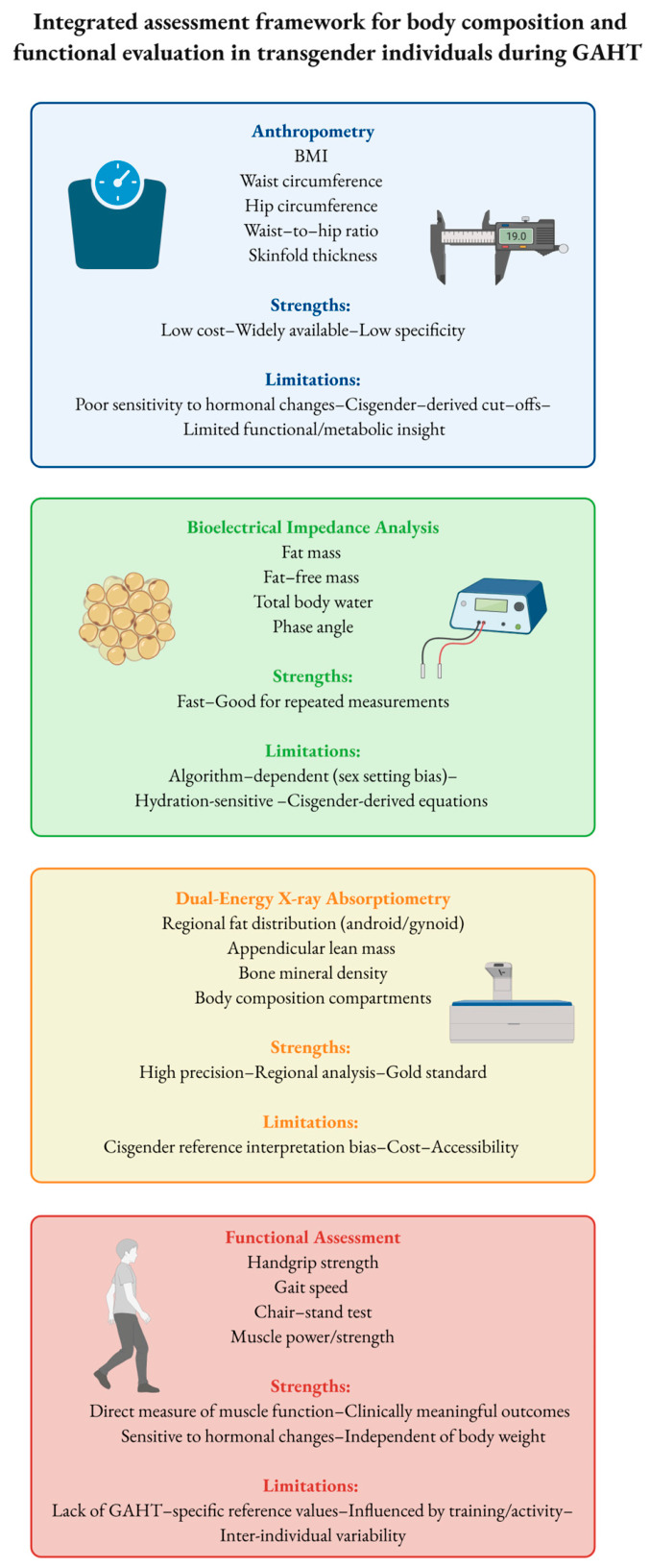
Reports the main methods for body composition and nutritional assessment in transgender and gender-diverse individuals undergoing GAHT.

Figure 1 summarizes the hierarchical framework of assessment tools, ranging from simple clinical measures (anthropometry and functional tests) to advanced techniques (BIA and DXA), highlighting their respective strengths, limitations, and interpretative challenges in the context of hormonally driven body composition changes.

## 5. Assessment in GAHT

### 5.1. Baseline Assessment Before or at GAHT Initiation

A comprehensive baseline assessment represents the foundation of nutritional and body composition monitoring in transgender and gender-diverse individuals initiating GAHT. Given the time-dependent nature of these adaptations, baseline evaluation is essential both to document pre-treatment status and to enable meaningful longitudinal interpretation [4,5,71]. Baseline assessment should move beyond isolated measures, such as body weight or BMI, and adopt a multidimensional approach, integrating four domains: anthropometry, body composition, functional capacity, and biochemical/metabolic profiling. Anthropometric evaluation should include body weight, height, waist and hip circumferences, as well as waist-to-hip ratio, with the optional inclusion of mid-upper-arm and calf circumferences when peripheral tissue status is relevant. These measures are primarily useful for within-subject monitoring, as cross-sectional interpretation is limited by the lack of transgender-specific reference standards [22,24,50]. Whenever feasible, body composition should be assessed using DXA, which provides robust characterization of fat mass, LBM, regional compartments, and bone mineral density. When DXA is not available, BIA may still offer useful longitudinal information, provided its limitations are acknowledged [21,22,50,72]. Standardization of measurement conditions (e.g., hydration status, fasting state, device consistency) is essential to ensure comparability over time.

Functional assessment should be systematically included. Handgrip strength is a simple and clinically meaningful indicator of muscle function, complementing compositional data and allowing early identification of reduced functional reserve. Additional tests, such as chair–stand or gait speed, may be considered in selected cases [12,21,32].

Biochemical assessment should include lipid profile, glucose-related indices, liver and renal function, hematologic parameters, and bone-related markers when indicated. These measures are essential for both endocrine safety and metabolic assessment [5,44,71].

Baseline evaluation should also include a structured assessment of dietary intake and physical activity. Psychosocial factors, including food insecurity, body image, and disordered eating, should be considered due to their impact on nutritional status and metabolic adaptation [18,44,45]. When feasible, standardized tools (e.g., dietary recalls or questionnaires) can improve reproducibility and support follow-up.

Overall, baseline assessment should be considered an individual reference point, serving as the basis for trajectory-based monitoring and personalized care.

### 5.2. Longitudinal Monitoring During GAHT

Longitudinal monitoring during GAHT should be viewed as a trajectory-based process, aimed at assessing whether physiological changes align with expected hormonal adaptation. In clinical practice, this involves repeated, standardized assessments to detect deviations in body composition, metabolic profile, and functional status [4,13,44]. Follow-up should be more frequent during the first 6–12 months of GAHT, when changes are most pronounced, with monitoring focused on LBM, fat mass, waist circumference, lipid profile, glucose metabolism, hematologic parameters, dietary intake, protein adequacy, and muscle function [4,5,13,21,22,27]. Thereafter, follow-up can be spaced according to clinical stability, prioritizing body composition trajectories, visceral adiposity, bone health, cardiometabolic risk, physical performance, exercise habits, and dietary sustainability [13,21,27,35,44]. Monitoring should consistently integrate anthropometry, body composition, functional measures, biochemical parameters, and lifestyle factors.

Anthropometric measures, including body weight, waist circumference, and waist-to-hip ratio, should be repeated using standardized protocols, with emphasis on tracking changes over time rather than relying on single values, particularly in the context of GAHT-induced remodeling [11,22]. When body composition techniques are available, methodological consistency is essential. DXA remains the reference method, while BIA may be used for within-subject monitoring, provided its limitations are acknowledged. Longitudinal data confirm that changes occur mainly within the first year and may plateau thereafter, supporting the need for consistent measurement intervals [13,21]. Although lean mass and fat mass changes often show partial stabilization after the first year, stabilization should not be interpreted as complete convergence toward cisgender reference patterns. Regional adiposity, visceral fat, bone remodeling, muscle strength, and cardiometabolic markers may remain intermediate or discordant, supporting continued within-subject monitoring rather than isolated comparison with cisgender thresholds [13,21,22,36,58].

Functional assessment should be routinely included. Handgrip strength is a practical and sensitive marker of functional adaptation and may detect changes not captured by compositional measures alone. Evidence indicates that strength and LBM do not always change in parallel, reinforcing the need for integrated assessment [30,31,32].

Biochemical monitoring should include lipid profile, glucose metabolism, liver enzymes, renal function, and hematologic parameters, supporting interpretation of metabolic trajectories. These variables may change independently of body composition, highlighting the need for comprehensive evaluation [27,44,49].

Longitudinal follow-up should also include regular assessment of dietary intake and physical activity. Exercise habits may influence body composition, muscle strength, and cardiometabolic risk during GAHT. Resistance training may support LBM accretion in TM and help preserve muscle mass and strength in TW. However, direct evidence from structured exercise interventions in transgender populations remains limited; therefore, recommendations should be individualized and adapted from broader exercise and clinical nutrition literature [12,30,31,32,44,45]. Overall, monitoring should prioritize within-subject changes over cross-sectional classification, enabling early identification of suboptimal adaptation and timely adjustment of clinical and nutritional strategies.

### 5.3. Practical Tools for Routine Clinical Use

The translation of current evidence into clinical practice requires a pragmatic framework that balances feasibility and clinical relevance. No single measurement is sufficient to capture the complexity of nutritional and metabolic adaptation during GAHT; therefore, monitoring relies on the integration of complementary tools interpreted longitudinally [4,44]. Table 2 summarizes a stepwise clinical framework. This model prioritizes simple and reproducible measures, such as anthropometry and handgrip strength, as the foundation of routine practice, while reserving DXA for baseline assessment and targeted follow-up [13,21]. A key principle is that clinically meaningful information arises from the combination of variables rather than isolated measures. For example, stable body weight with increasing waist circumference, declining strength, or worsening metabolic markers may indicate unfavorable adaptation, whereas improved function despite modest compositional change may reflect beneficial physiological adaptation. Clinical interpretation should therefore rely on trajectory analysis and pattern recognition rather than single thresholds. This approach enables early identification of discordant adaptations and supports timely intervention.

### 5.4. Nutrition-Centered Priorities

GAHT creates a dynamic metabolic environment in which nutritional needs change over time, driven by alterations in body composition, energy expenditure, and tissue function. Nutritional management should therefore align dietary intake with physiological adaptation. A key challenge is the alignment between hormonal adaptation and dietary intake, as mismatch may lead to suboptimal body composition, impaired function, or cardiometabolic risk. Evidence suggests that dietary variability contributes significantly to interindividual differences in metabolic response [18,20,44,71].

#### 5.4.1. Transgender Men

In TM, priorities focus on supporting anabolic adaptation while maintaining metabolic balance. Adequate energy and protein intake are essential for muscle accretion, while excessive intake may promote visceral fat accumulation. Insufficient protein may blunt anabolic response, whereas excess calories, particularly from low-quality diets, may promote adverse fat redistribution. Strategies should prioritize adequate protein intake, energy alignment, and dietary quality [31,46,47,72].

#### 5.4.2. Transgender Women

In TW, priorities focus on preserving LBM and preventing excess adiposity in the context of reduced anabolic signaling. Reduced energy expenditure and anabolic drive create a context where both over- and under-nutrition may negatively affect outcomes. Preservation of muscle mass requires adequate protein intake, regular distribution, and resistance exercise [42,48].

#### 5.4.3. Beyond Macronutrients

Nutritional status during GAHT is also influenced by behavioral and psychosocial factors, including dietary patterns, food access, eating behavior, and body image-related aspects. These factors may evolve over time and interact with physiological adaptation, contributing to variability in metabolic and compositional outcomes [18,19], reinforcing the need for individualized nutritional strategies integrated into gender-affirming care pathways. Based on current evidence, pragmatic and physiology-informed macronutrient priorities are summarized in Table 3, providing a clinically applicable framework for nutritional management during GAHT.

In addition to macronutrients, selected micronutrients may be clinically relevant during GAHT, particularly in relation to skeletal health, hematologic adaptation, dietary adequacy, restrictive eating patterns, and comorbidities. Because micronutrient requirements and risks may differ between transgender men and transgender women, especially for iron metabolism and bone health, assessment should be individualized according to hormone regimen, treatment phase, dietary intake, laboratory parameters, and clinical phenotype. The key considerations are summarized in Table 4.

Bone-related nutrients, particularly vitamin D and calcium, are a key area of clinical relevance. Transgender women may present with reduced bone mineral density at baseline and show variable skeletal responses during GAHT, underscoring the need for adequate intake and monitoring of these nutrients [12,34,35]. In transgender men, testosterone-related changes in bone turnover further support the importance of nutritional strategies for skeletal health.

Iron metabolism is also affected by GAHT. Testosterone therapy in transgender men increases erythropoiesis and may raise iron requirements or alter iron balance, whereas transgender women may experience a reduction in erythropoietic drive over time [27]. These changes highlight the need for individualized monitoring of hematologic and iron-related status rather than routine supplementation.

Nutrients involved in cardiometabolic regulation, such as omega-3 fatty acids, may also be relevant in light of GAHT-associated changes in lipid profile [27,43]. Although evidence remains indirect, dietary patterns rich in unsaturated fats may support improved metabolic outcomes.

Overall, micronutrient management during GAHT should be individualized and embedded within comprehensive nutritional assessment, integrating dietary intake, biochemical markers, and clinical context. Instead of relying on universal supplementation strategies, clinicians should focus on targeted identification and correction of deficiencies to support optimal metabolic and functional outcomes.

## 6. Conclusions

GAHT induces complex, time-dependent, and tissue-specific changes in body composition, metabolism, and functional capacity that are not adequately captured by conventional nutritional assessment models. These adaptations extend beyond simple shifts in fat and LBM, involving coordinated changes in energy expenditure, adipose distribution, muscle function, and metabolic regulation. A central finding is that nutritional assessment in transgender individuals requires a multidimensional and longitudinal framework, integrating anthropometry, body composition techniques, functional measures, and biochemical markers. Reliance on single indicators, particularly BMI, may lead to misclassification and underestimation of clinically relevant changes during hormonal transition. Metabolic trajectories differ between transgender men and transgender women and are phase-dependent, resulting in distinct nutritional priorities. In transgender men, the focus is on supporting anabolic adaptation while maintaining metabolic balance, whereas in transgender women the priority is to preserve LBM and limit excess adiposity. These differences highlight the limitations of applying cisgender-based nutritional models to GAHT populations.

From a clinical perspective, optimal management requires a trajectory-based and nutrition-centered approach, in which dietary strategies are continuously aligned with physiological changes induced by hormonal treatment. Mismatches between energy intake, body composition remodeling, and metabolic adaptation appear to be key drivers of suboptimal outcomes, emphasizing the need for individualized and phase-specific interventions. Despite growing evidence, important gaps remain, particularly the absence of transgender-specific reference standards for body composition, functional assessment, and metabolic risk stratification. Future research should focus on longitudinal, well-characterized cohorts integrating dietary intake, body composition, metabolic outcomes, and functional performance.

Overall, integrating nutritional assessment into gender-affirming care is essential. A multidisciplinary, individualized, and physiology-informed approach, with nutrition professionals playing a central role, is required. Moving from static, sex-based frameworks toward dynamic, trajectory-oriented models represents a necessary step forward in the clinical management of individuals undergoing GAHT.

## 7. Limitations and Future Research Directions

Current evidence on nutritional and body composition monitoring during GAHT remains limited by the predominance of observational studies, heterogeneous methodologies, small cohorts, variable hormone regimens, short follow-up duration, and the lack of validated transgender-specific reference standards [15,22,36,55,58,68,75]. In addition, the increasing use of modern metabolic therapies further reinforces the need to interpret weight change through body composition analysis. For example, the systematic review and meta-analysis by Sawicka-Gutaj et al. showed that GLP-1 receptor agonists and dual GLP-1/GIP agonists are associated with clinically relevant weight loss, with greater reductions in fat mass and visceral adiposity than in lean mass [76]. Although these findings are not specific to transgender populations, they support the broader principle that nutritional monitoring should distinguish weight change from changes in fat mass, visceral adiposity, and lean mass, particularly when metabolic therapies coexist with GAHT.

Artificial intelligence and machine learning may eventually contribute to this field by integrating multidimensional longitudinal data and supporting more individualized interpretation of body composition and metabolic trajectories [28,77]. However, their current applicability in gender-affirming nutritional care remains theoretical. The lack of large, representative, and externally validated datasets, together with the risk of algorithmic bias when models are trained on cisgender-dominant data, prevents immediate clinical implementation [78,79,80]. Therefore, AI should be considered a future research direction rather than a current clinical tool. Until such evidence becomes available, nutritional care during GAHT should rely on individualized assessment, repeated within-subject monitoring, and integration of body composition, biochemical, functional, dietary, and psychosocial data.

## Figures and Tables

**Table 1:** Summary of GAHT-related body composition, metabolic, functional, and skeletal effects in transgender men and transgender women.

Domain	TM + Testosterone	TW + Estrogen/Anti-Androgen	Interpretation	References
Lean body mass/fat-free mass	Increased; meta-analytic estimates report approximately +3.9 to +4.98 kg within the first year	Reduced, with meta-analytic estimates of approximately −2.4 kg within the first year	One of the most consistent GAHT-related changes; should be interpreted with muscle function	[10,11,12,13,21,22]
Fat mass	Generally reduced; meta-analytic estimates report approximately −2.6 kg, although VAT may increase in some studies	Generally increased; meta-analytic estimates report approximately +3.0 kg, with redistribution toward subcutaneous/gynoid depots	GAHT reshapes adiposity instead of simply increasing or decreasing total fat mass	[10,11,13,14,21,22,23,24,25]
Visceral and ectopic fat	Possible VAT increase despite reduced total fat mass	Variable VAT response; hepatic fat may change independently of total adiposity	Regional fat depots may be more clinically informative than body weight or BMI	[13,14,16,23,24,25]
Resting energy expenditure/substrate metabolism	May increase, partly reflecting higher metabolically active lean tissue	May decrease, partly reflecting reduced FFM; direct hormonal effects may also contribute	Energy requirements should be reassessed longitudinally	[8,15,16,17,26]
Glucose–insulin homeostasis	Heterogeneous findings; insulin sensitivity may remain stable or change depending on baseline status	Inconsistent findings, with reduced insulin sensitivity or minimal changes reported	Responses depend on age, baseline metabolic status, treatment duration, and lifestyle	[15,16,26,27]
Lipid profile and cardiovascular markers	HDL often decreases; LDL, triglycerides, blood pressure, and hematocrit may vary	Triglycerides and pro-thrombotic markers may increase depending on regimen and risk profile	Cardiometabolic monitoring should not rely on BMI or anthropometry alone	[27,28,29]
Muscle strength and physical function	Strength may increase, but not always proportionally to LBM	Strength and power may decline, with heterogeneous upper- and lower-body responses	Functional assessment should complement body composition	[12,30,31,32,33]
Bone health	BMD is generally maintained or increased, with possible site-specific variability	Baseline BMD may be suboptimal; response depends on age, adherence, and hormone exposure	Bone assessment should consider treatment adherence, vitamin D/calcium intake, and risk factors	[33,34,35,36,37,38,39,40,41]
Nutritional implications	Support lean mass accretion, protein adequacy, energy alignment, and cardiometabolic risk reduction	Preserve lean mass, ensure protein adequacy, encourage resistance exercise, and monitor cardiometabolic risk	Nutritional care should be individualized and longitudinal	[4,5,18,27,42,43,44,45,46,47,48]

Note. The table summarizes the direction of the main GAHT-related changes reported in the available literature. Given the heterogeneity of study design, population characteristics, hormone regimens, and follow-up duration, findings should be interpreted as overall trends rather than definitive effect estimates. Abbreviations: GAHT = gender-affirming hormone therapy; TM = transgender men; TW = transgender women; LBM = lean body mass; FFM = fat-free mass; VAT = visceral adipose tissue; BMI = body mass index; HDL = high-density lipoprotein; LDL = low-density lipoprotein; BMD = bone mineral density.

**Table 2:** Practical, stepwise monitoring of nutritional and body composition status during GAHT.

Domain	Tool	When	Clinical Meaning	Clinical Signals	Key References
Anthropometry	Body weight	Every visit	Overall trend	Stable weight with hidden composition changes	[13,22]
	WC	Every visit	Central adiposity	↑ waist despite stable weight → visceral fat increase	[14,16]
	WHR	Every visit	Fat redistribution	Incomplete shift toward affirmed phenotype	[21,22]
	Arm circumference	Selected	Peripheral muscle proxy	↓ arm circ → muscle loss	[59]
	Calf circumference	Selected	Muscle reserve	↓ calf circ → sarcopenic risk	[59,60]
Functional	Handgrip strength	Baseline + follow-up	Muscle function	↓ strength despite stable LBM	[12,30]
	Chair–stand/gait speed	Selected	Physical performance	Reduced performance → early functional decline	[30]
Body composition	BIA	Serial follow-up	FM, FFM trends, phase angle	↓ phase angle → reduced cellular integrity	[50,51,52]
	Skinfolds	Selected	Subcutaneous fat distribution	Discordance with waist → altered fat pattern	[61,62,63]
Advanced	DXA	Baseline + selected follow-up	FM, LBM, appendicular mass, BMD	Lean loss, VAT shift, bone changes	[10,11,13,14,35]
Biochemical	Lipid profile	Routine	Cardiometabolic risk	↑ TG/↓ HDL	[27,49]
	Glucose metabolism	Routine	Insulin sensitivity	↑ glucose/insulin	[15,26]
	Liver enzymes	Routine	Hepatic involvement	↑ ALT/AST	[16]
	Hematology	Routine	Therapy response	↑ hematocrit (TM)	[27]
Nutrition & lifestyle	Dietary intake	Every visit	Energy/protein balance	Mismatch intake vs. adaptation	[18,44]
	Physical activity	Every visit	Muscle/metabolic modulation	Low activity → poor adaptation	[30,32]

Abbreviations: WC = waist circumference; WHR = waist-to-hip ratio; LBM = lean body mass; BIA = bioelectrical impedance analysis; DXA = dual-energy x-ray absorptiometry; FM = fat mass; FFM = fat free mass; BMD = bone mineral density; VAT = visceral adipose tissue; TG = triglycerides; HDL = high-density lipoprotein; TM = transgender men.

**Table 3:** Pragmatic macronutrient priorities during GAHT.

Macronutrient	Population	Clinical Relevance During GAHT	Potential Risk	Practical Recommendation	Key References
Energy	TM	Increased energy expenditure during anabolic phase	Inadequate intake → limited muscle accretion; excess → visceral fat gain	Adjust energy intake dynamically based on body composition and metabolic trajectory	[18,44]
	TW	Reduced energy expenditure	Positive energy balance → fat accumulation	Reassess caloric intake; avoid maintaining pre-GAHT intake without adjustment	[18,44]
Protein	TM	Supports muscle protein synthesis and androgen-mediated anabolic response	Low intake → suboptimal lean mass gain	~1.2–1.6 g/kg/day (higher if training); distribute across ≥3 meals (~0.3–0.4 g/kg/meal)	[7,42]
	TW	Preserves lean mass and function	Loss of muscle mass and functional decline	~1.0–1.3 g/kg/day; prioritize regular intake across meals; ≥3 meals/day with adequate protein per meal	[31,42]
Fat	TM & TW	Modulates lipid profile and cardiometabolic risk during GAHT	Dyslipidemia (↓ HDL, ↑ LDL/TG)	Limit saturated fats; increase MUFA/PUFA; favor Mediterranean-type pattern	[27,43]
Carbohydrates	TM & TW	Contribute to metabolic control and insulin sensitivity	Excess refined CHO → metabolic impairment	Prefer complex CHO, fiber-rich foods	[15,16]

Note. These values represent pragmatic, physiology-informed ranges derived from available evidence in transgender populations and the broader clinical nutrition literature and should be individualized according to age, treatment phase, body composition trajectory, physical activity level, comorbidities, and dietary assessment. Abbreviations: GAHT = gender-affirming hormone therapy; TM = transgender men; TW = transgender women; HDL = high-density lipoprotein; LDL = low-density lipoprotein; TG = triglycerides; MUFA = monounsaturated fatty acids; PUFA = polyunsaturated fatty acids; CHO = carbohydrates.

**Table 4:** Micronutrient considerations during GAHT according to population-specific clinical relevance, potential risk, and practical recommendations.

Population	Micronutrient	Clinical Relevance	Potential Risk	Practical Recommendation	References
TM	Calcium	Bone support during testosterone-related skeletal adaptation	Low intake, low vitamin D, low BMI, restrictive eating, or poor GAHT adherence	Aim for age-appropriate intake, generally 1000–1200 mg/day, preferably from diet	[12,34,35]
TW		Bone support is relevant because baseline BMD may be lower and depends on adequate estrogen exposure	Low BMD, inadequate estrogen exposure, gonadectomy without adequate replacement, low BMI, or restrictive eating	Aim for age-appropriate intake, generally 1000–1200 mg/day; assess more closely in those at bone risk	[12,34,37]
TM	Vitamin D	Supports bone and muscle function during masculinizing GAHT	Deficiency may worsen skeletal or muscle outcomes, especially with obesity, low sun exposure, or restrictive eating	Reference intake of 15–20 µg/day, 600–800 IU/day, according to age; check 25-OH vitamin D when risk factors are present	[12,34,37]
TW		Supports bone and muscle function, especially with low BMD or reduced LBM	Deficiency may worsen bone vulnerability, particularly with inadequate estrogen exposure or poor adherence	Reference intake of 15–20 µg/day, 600–800 IU/day, according to age; check 25-OH vitamin D in those with bone-risk factors	[12,34,37]
TM	Magnesium	Relevant for muscle function, glucose metabolism, cardiovascular regulation, and bone health	Low intake may worsen metabolic vulnerability; evidence in GAHT is indirect	Reference intake of 400–420 mg/day when male-based values are used; prioritize magnesium-rich foods and supplement only if indicated	[18,44,45,73]
TW		Relevant for bone, muscle, glucose metabolism, and cardiometabolic health	Low intake may be relevant with poor diet quality, restrictive eating, low BMD, or metabolic risk; GAHT-specific evidence is limited	Reference intake of 310–320 mg/day when female-based values are used; prioritize magnesium-rich foods and supplement only if indicated	[18,44,45,73]
TM	Iron	Testosterone may increase erythropoiesis, hemoglobin, and hematocrit	Increased erythropoietic demand, but also risk of excessive hematocrit	Adult reference intake is commonly 8–10 mg/day; monitor ferritin, hemoglobin, and hematocrit; supplement only if deficiency is documented	[27,74]
TW		Androgen suppression may reduce erythropoietic drive over time	Routine supplementation may be inappropriate; deficiency remains possible with low intake, bleeding, or clinical conditions	Reference intake, depending on menstruation and clinical status, of 18 mg/day if menstruating, 8–10 mg/day if non-menstruating; supplement only if deficiency is confirmed	[27,74]
TM & TW	Vitamin B12/folate	Relevant for hematologic function and diet-related vulnerability	Risk with vegan diets, restrictive eating, food insecurity, selective eating, or malabsorption	Reference intake of vitamin B12 2.4 µg/day; folate 400 µg DFE/day; assess labs when clinically indicated	[18,20,71]
TM & TW	Other micronutrients	Zinc, iodine, selenium, and others may be relevant depending on diet quality and comorbidities	Deficiencies may be under-recognized with poor diet quality, restrictive eating, or malabsorption	Routine broad screening is not supported; use targeted assessment based on diet, phenotype, and labs	[18,44,45]

Note. Transgender-specific quantitative micronutrient requirements are not currently available. Therefore, reference intakes should be interpreted according to age, physiology, dietary pattern, clinical risk, laboratory values, and treatment context rather than applied rigidly according to binary sex categories. Routine broad supplementation is not recommended without documented deficiency or specific clinical indication. Abbreviations: GAHT = gender-affirming hormone therapy; TM = transgender men; TW = transgender women; BMD = bone mineral density; BMI = body mass index; LBM = lean body mass; 25-OH vitamin D = 25-hydroxyvitamin D; DFE = dietary folate equivalents.

## Data Availability

No new data were created or analyzed in this study. Data sharing is not applicable to this article.
